# Chronic Ulnar-Sided Wrist Pain Caused by a Rare Glomangioma: A Case Report

**DOI:** 10.7759/cureus.115706

**Published:** 2026-09-03

**Authors:** Sierra A Ziegler, Corey R Zimmer, Brian S Ziegler

**Affiliations:** 1 Medicine, Lake Erie College of Osteopathic Medicine, Erie, USA; 2 Orthopaedic Surgery, The Orthopaedic Institute, Rockledge, USA

**Keywords:** chronic wrist pain, extradigital glomus tumor, glomangioma, glomus tumor rare locations, soft-tissue tumor, ulnar sided wrist pain, wrist mass

## Abstract

Glomus tumors are benign neoplasms typically found in the fingers and usually present with localized pain, swelling, and cold sensitivity. Extradigital glomus tumors are rare, and their diagnosis is often delayed due to atypical location and lack of cold sensitivity. In this article, we report a unique case of an extradigital glomangioma, a subset of glomus tumors, masquerading as more common ulnar-sided wrist pathology, such as a triangular fibrocartilage complex tear or extensor carpi ulnaris tendinitis. The patient presented with a two-year history of gradually worsening ulnar-sided wrist pain with no history of trauma. Examination revealed localized tenderness on the ulnar wrist without a palpable mass. X-rays were normal, and the pain was unresponsive to nonsteroidal anti-inflammatory medication and cortisone injection. Magnetic resonance imaging revealed a subcentimeter mass in the subcutaneous fat on the ulnar side of the wrist. The lesion was bright on T2-weighted and IV gadolinium-enhanced sequences. Excisional biopsy allowed the pathological diagnosis of benign glomangioma to be established. The patient reported immediate, complete resolution of pain postoperatively. This case underscores the importance of advanced imaging, clinical vigilance, and surgical excision for this rare tumor.

## Introduction

Chronic ulnar-sided wrist pain is a common presentation in orthopedic clinics. Frequent causes include degenerative joint disease, tendon pathology, or triangular fibrocartilage complex tears associated with degenerative conditions or traumatic injuries [1]. Most ulnar-sided wrist pain can be attributed to benign musculoskeletal conditions and is managed conservatively; however, persistent pain that fails to respond to standard therapies should prompt consideration of more uncommon etiologies.

This case highlights an unusual presentation of a variant of a glomus tumor on the ulnar side of the wrist, which masqueraded as more common wrist pathology. A glomus tumor is a benign neoplasm arising from the glomus body, a thermoregulatory arteriovenous shunt. The tumor typically presents with localized pain, lesion-associated tenderness, and cold sensitivity. While glomus tumors are most commonly found in the fingers, extradigital presentations can also occur. Diagnosis of an extradigital glomus tumor can be delayed due to the atypical location and lack of cold sensitivity [2-4].

## Case presentation

A 72-year-old right-hand-dominant male presented to the orthopedic office with a two-year history of gradually worsening left wrist pain. He reported no history of trauma. Over the preceding eight months, the pain had become severe and constant. It was localized to the ulnar aspect of the wrist and described as sharp, with radiation along the side of the wrist. The discomfort disrupted his sleep and worsened with wrist use. He denied numbness, tingling, or systemic symptoms.

On physical examination, the patient exhibited exquisite tenderness over the dorsoulnar wrist at the base of the fifth metacarpal. There was no palpable mass or visible swelling, and he had full active range of motion. Initial evaluation included plain radiographs, which demonstrated only mild degenerative changes in the left wrist without evidence of acute fracture or dislocation (Figure 1). 

**Figure 1:**
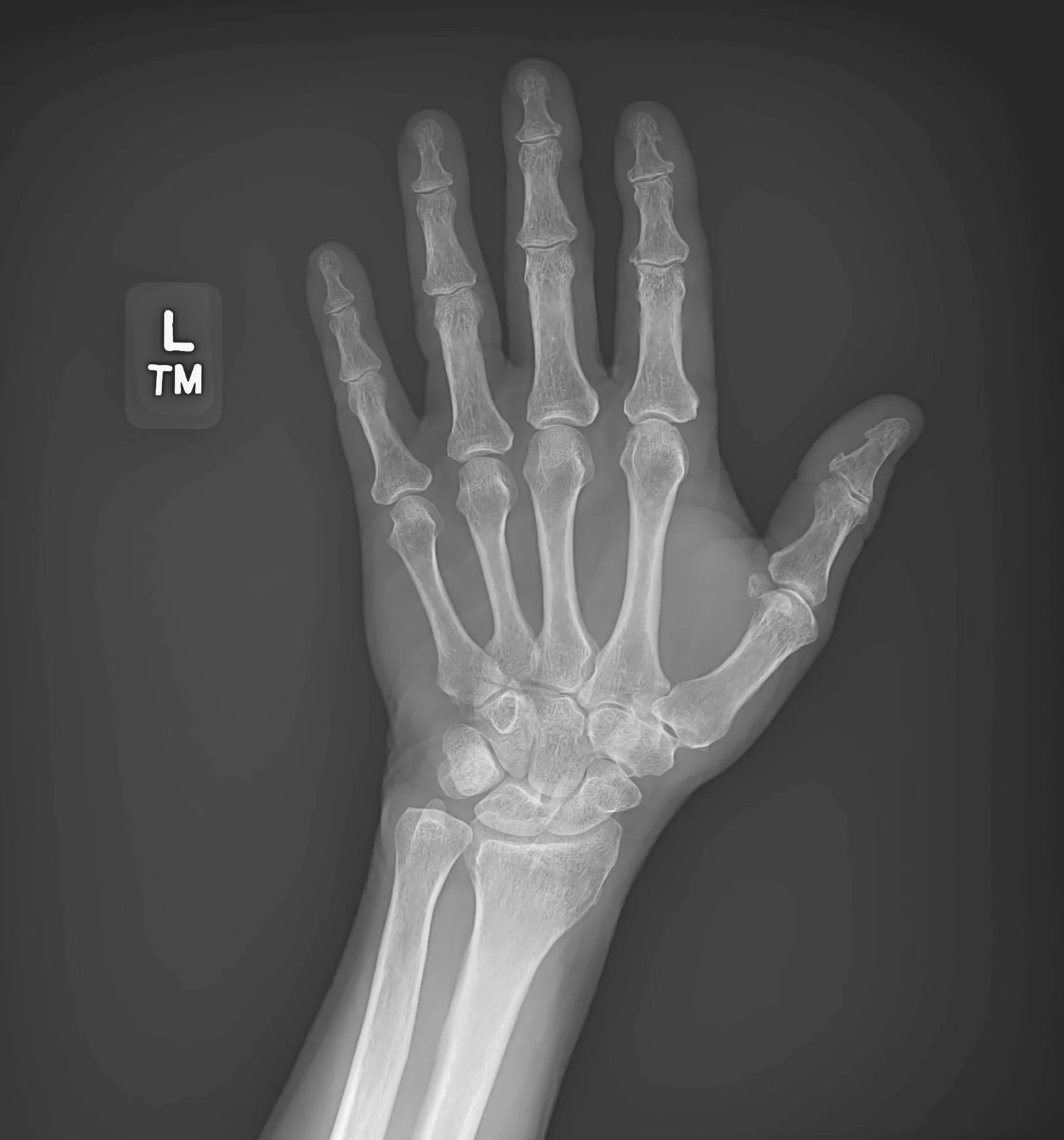
Anteroposterior X ray of the left hand demonstrating mild degenerative changes with no other abnormalities.

A wrist brace, NSAIDs, and an intra-articular corticosteroid injection were tried but failed to provide relief. Due to persistent symptoms, he underwent a magnetic resonance imaging (MRI) arthrogram (Figures 2, 3). This revealed a 0.6 x 0.6 x 0.7 cm lesion on T1 imaging within the subcutaneous fat on the ulnar side of the wrist. The radiologic differential diagnosis included a ganglion cyst or a prominent vein, and further imaging with intravenous (IV) contrast was recommended for clarification. 

**Figure 2:**
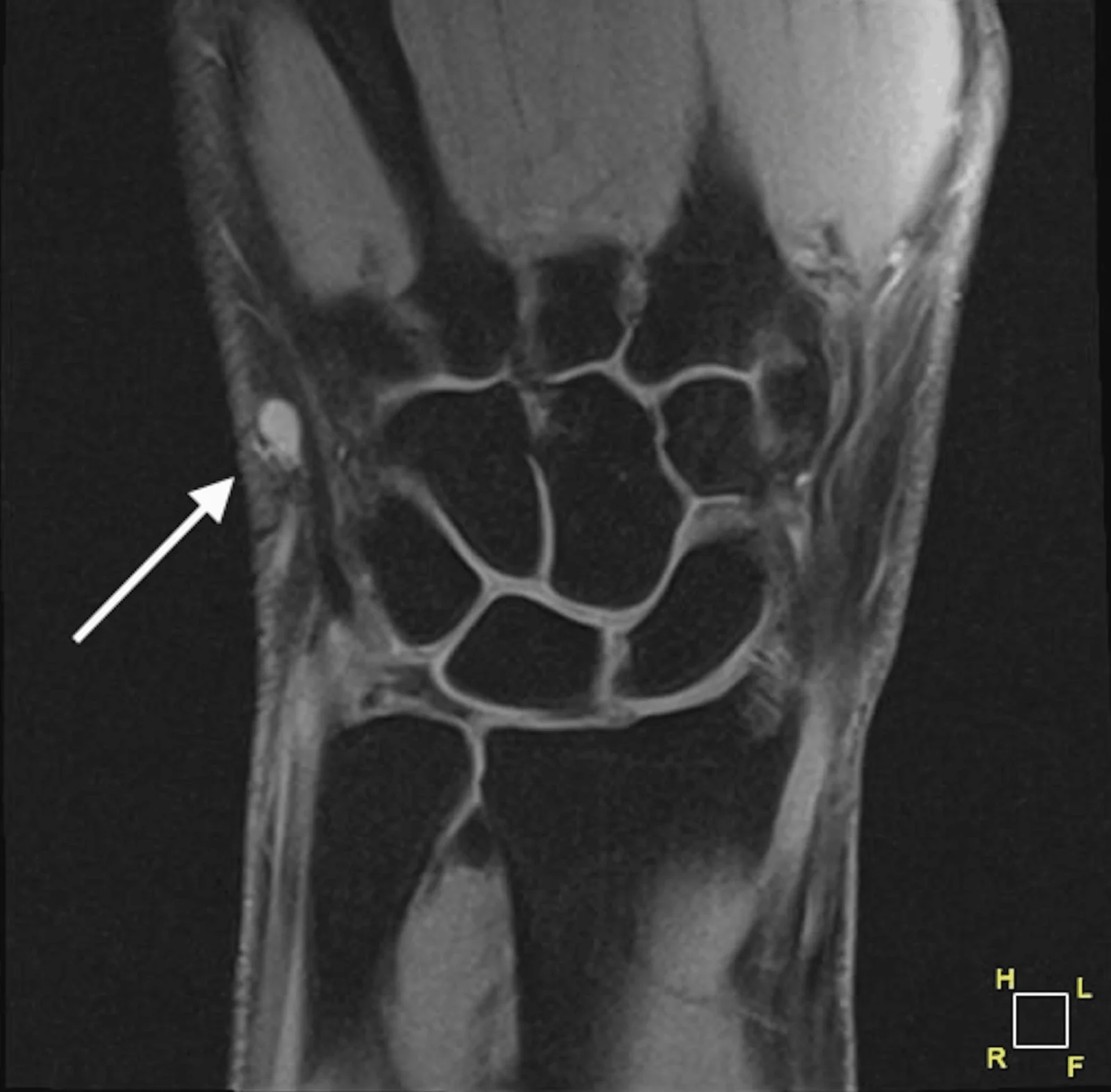
Pre-arthrogram coronal T1 image. H = head; F = foot; R = right; L = left.

**Figure 3:**
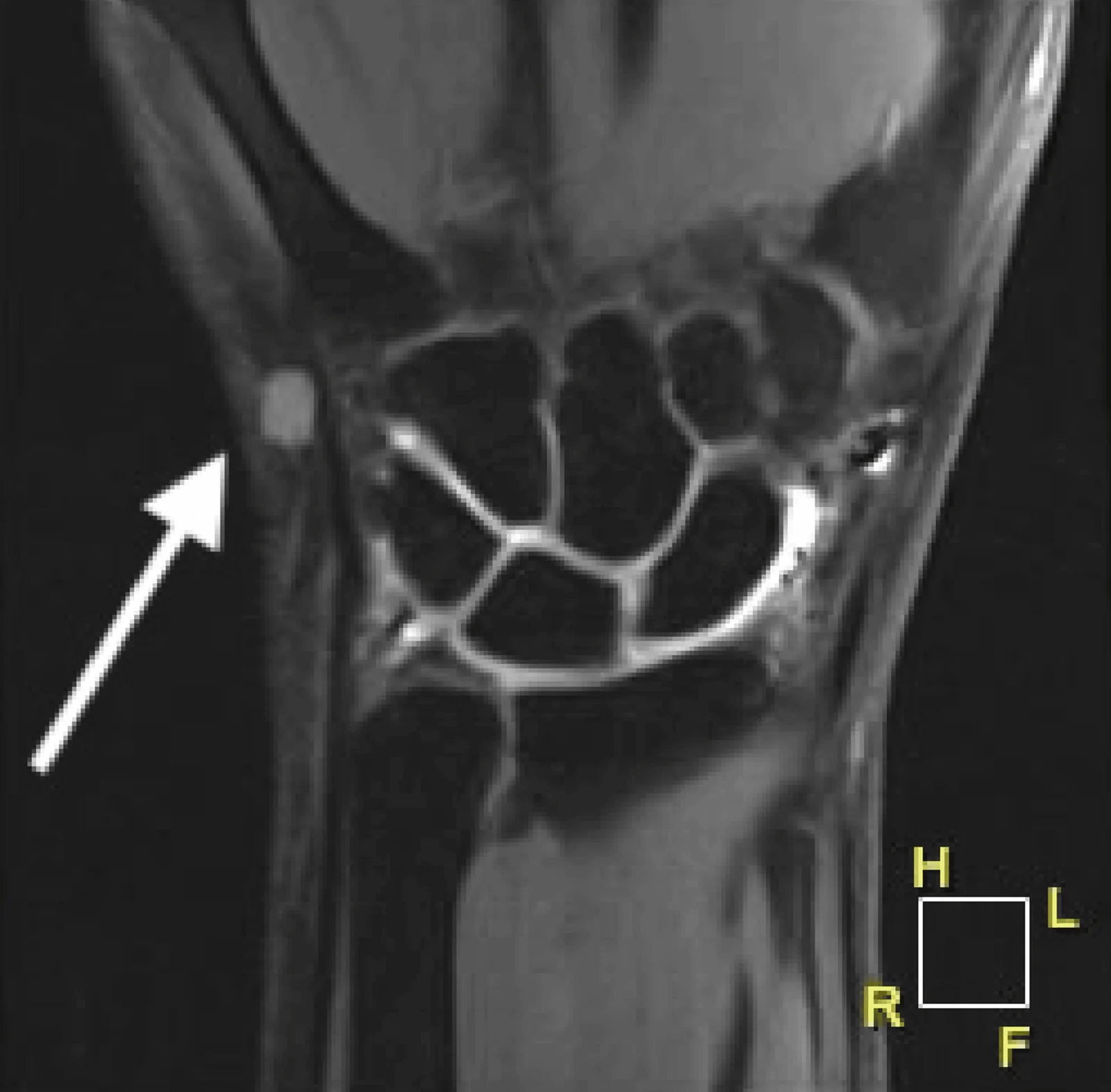
T1 image following an intra-articular gadolinium arthrogram.

Follow-up MRI with IV gadolinium contrast demonstrated that the lesion was hyperintense on precontrast T2 images and postcontrast T1 images (Figures 4-6). It remained stable in size. The findings raised concern for a small, well-defined soft tissue tumor. A computed tomography (CT) scan of the wrist further characterized the lesion as a subcentimeter, noncalcified soft tissue nodule within the subcutaneous fat at the level of the distal carpal row, again without defining features to differentiate between benign and malignant processes (Figure 7). The radiographic differential diagnosis for this mass is broad and includes tenosynovial giant cell tumor, nerve sheath tumors, synovial sarcoma, and other sarcomas. Enhancement with gadolinium makes a ganglion cyst unlikely. 

**Figure 4:**
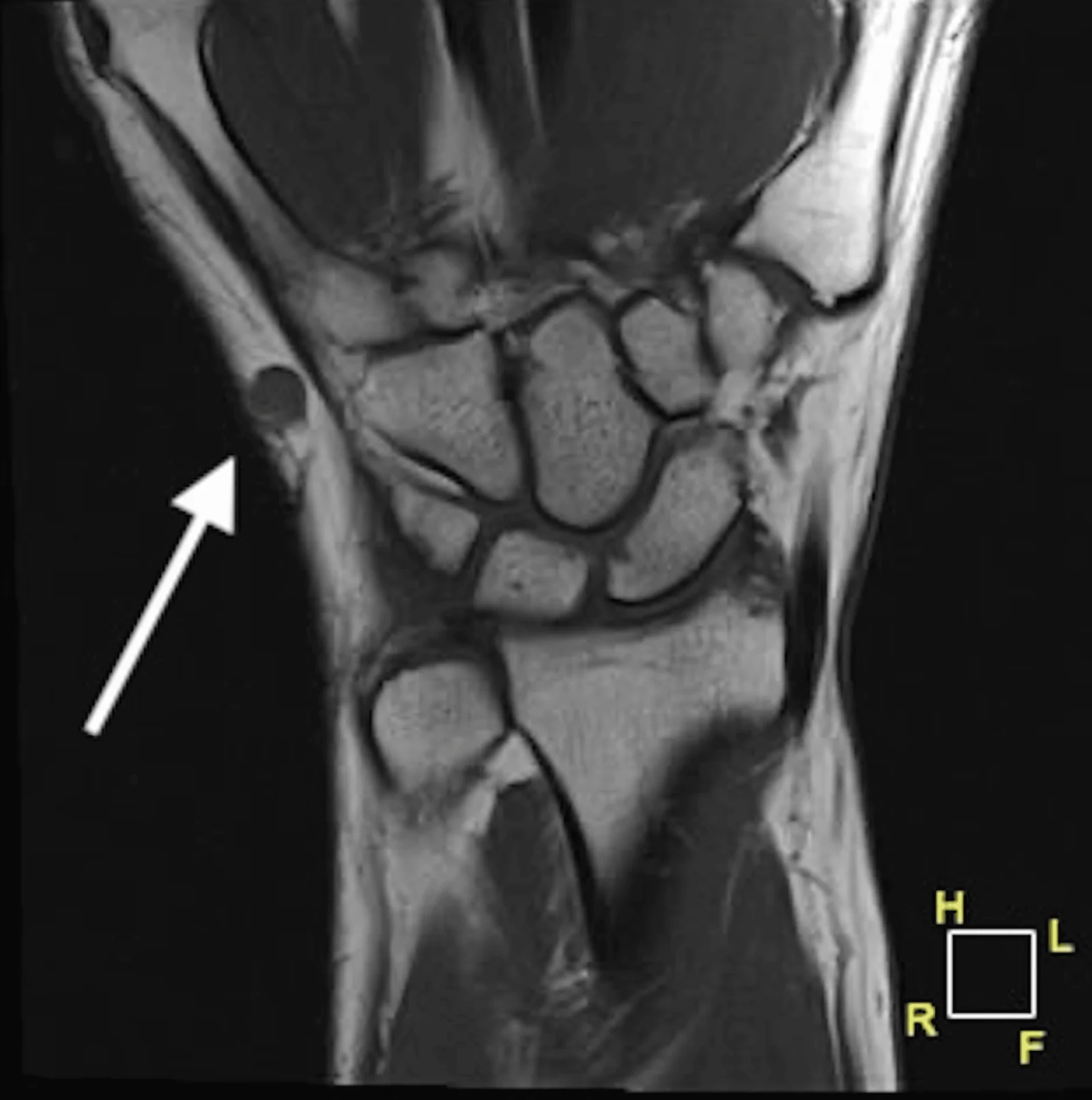
Pre-IV gadolinium contrast coronal T1 image revealing a lesion within the subcutaneous fat on the ulnar side of the wrist.

**Figure 5:**
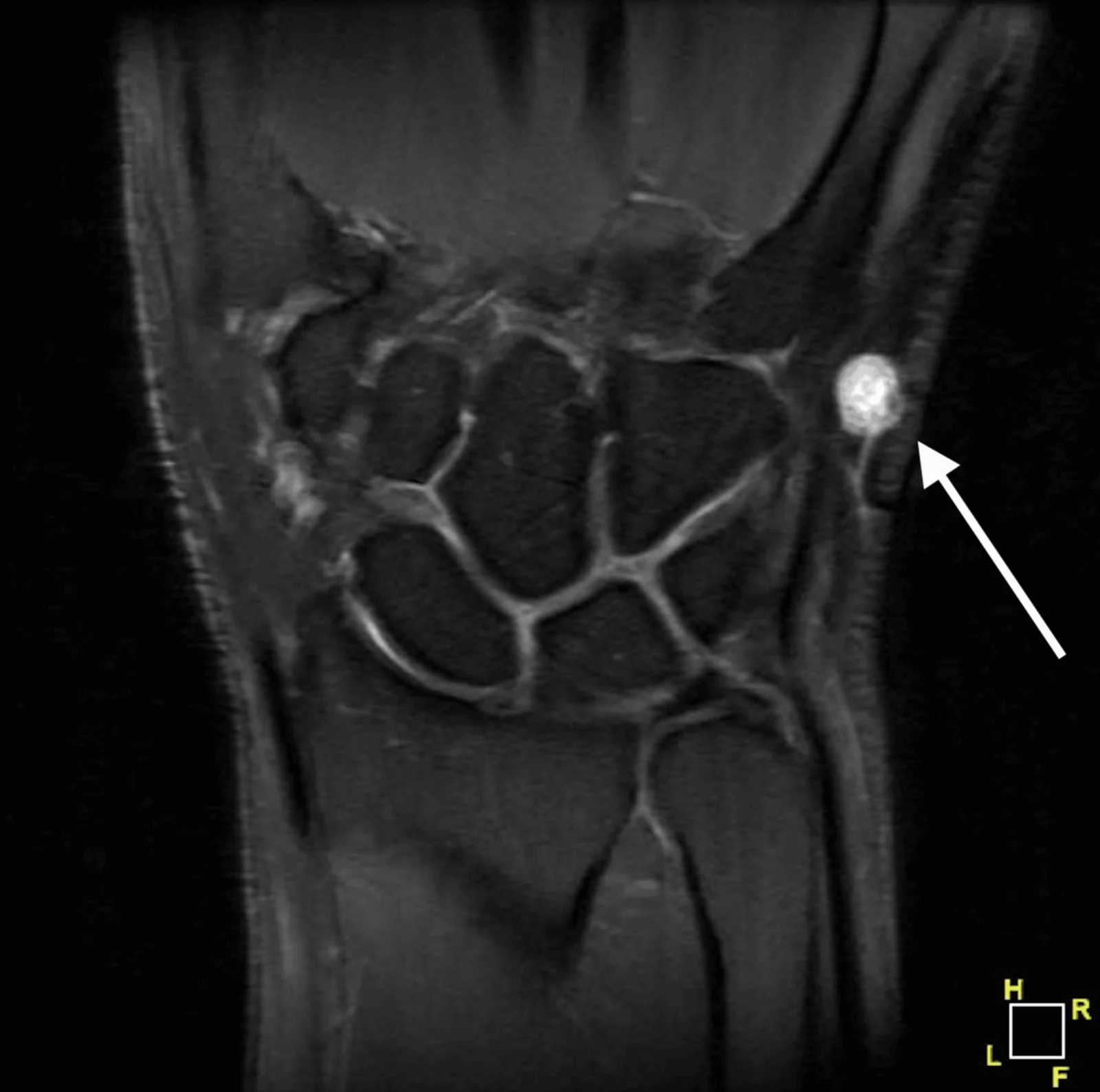
Precontrast coronal T2 image demonstrating a hyperintense subcentimeter nodule.

**Figure 6:**
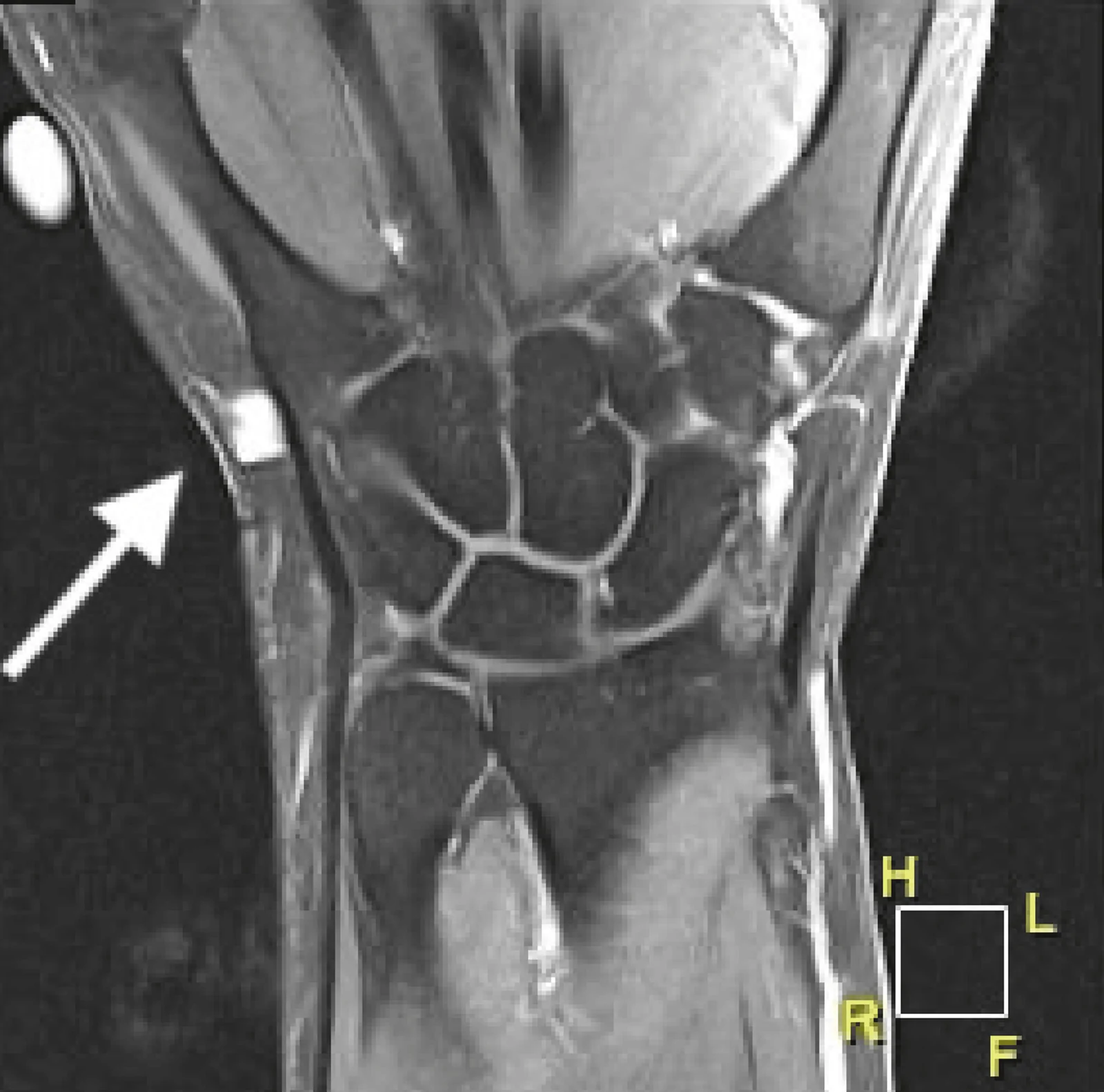
T1 image following IV gadolinium contrast administration.

**Figure 7:**
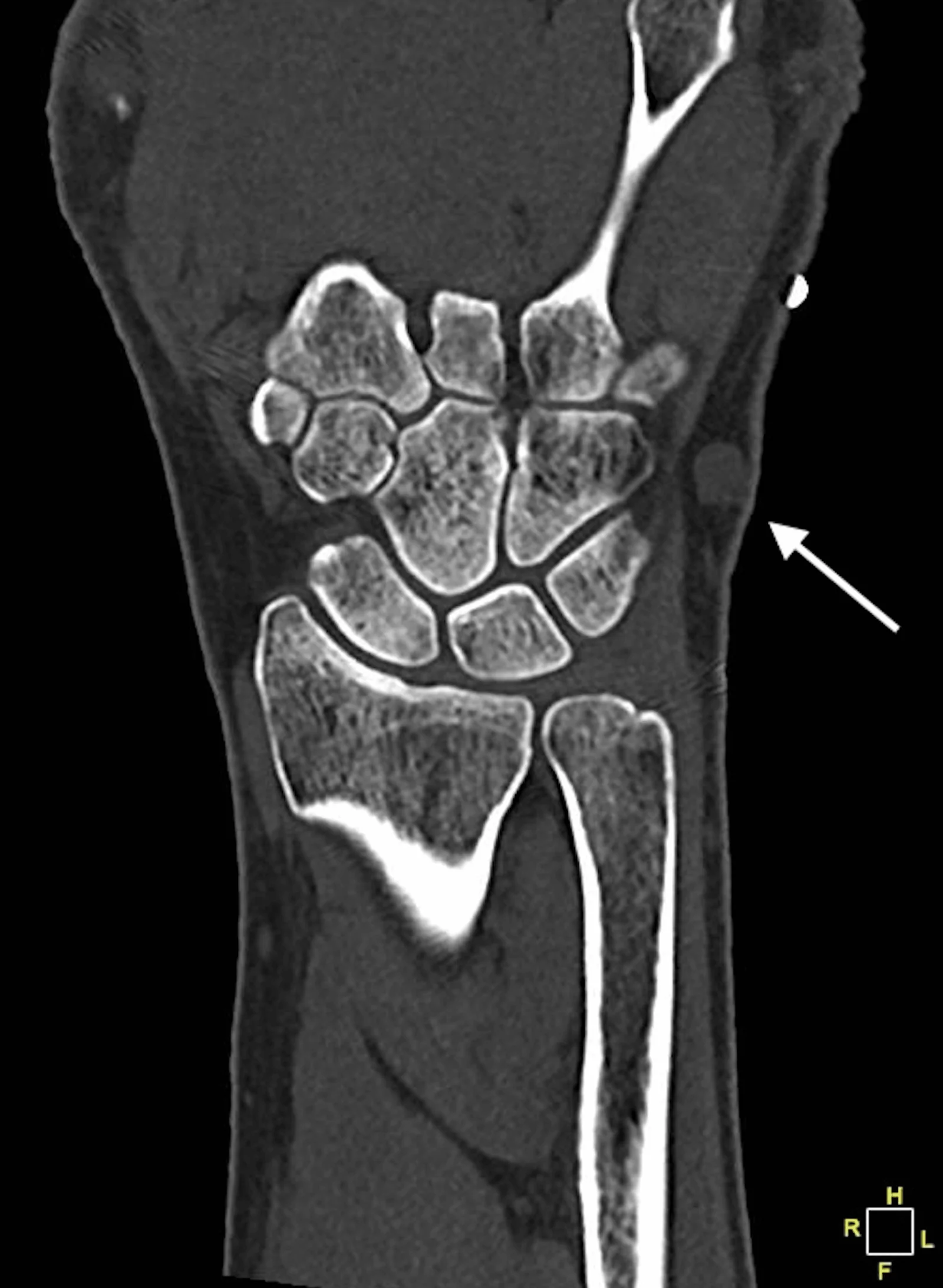
CT coronal image of the left wrist.

Since the lesion was less than 3 cm in size and superficial in location without abutment of a nerve or artery, the decision was made to proceed with excisional biopsy through a small incision. The patient was marked at the point of maximal tenderness in the holding area. A 1.5-cm longitudinal incision was made directly over the preoperative marking. A mildly protuberant, vascular mass was immediately identified. Careful dissection around the periphery resulted in complete excision of the mass. Pathology revealed a glomangioma, showing dilated vascular spaces surrounded by small, uniform polygonal glomus cells with eosinophilic cytoplasm and central nuclei, features characteristic of this tumor type (Figure 8) [5]. 

**Figure 8:**
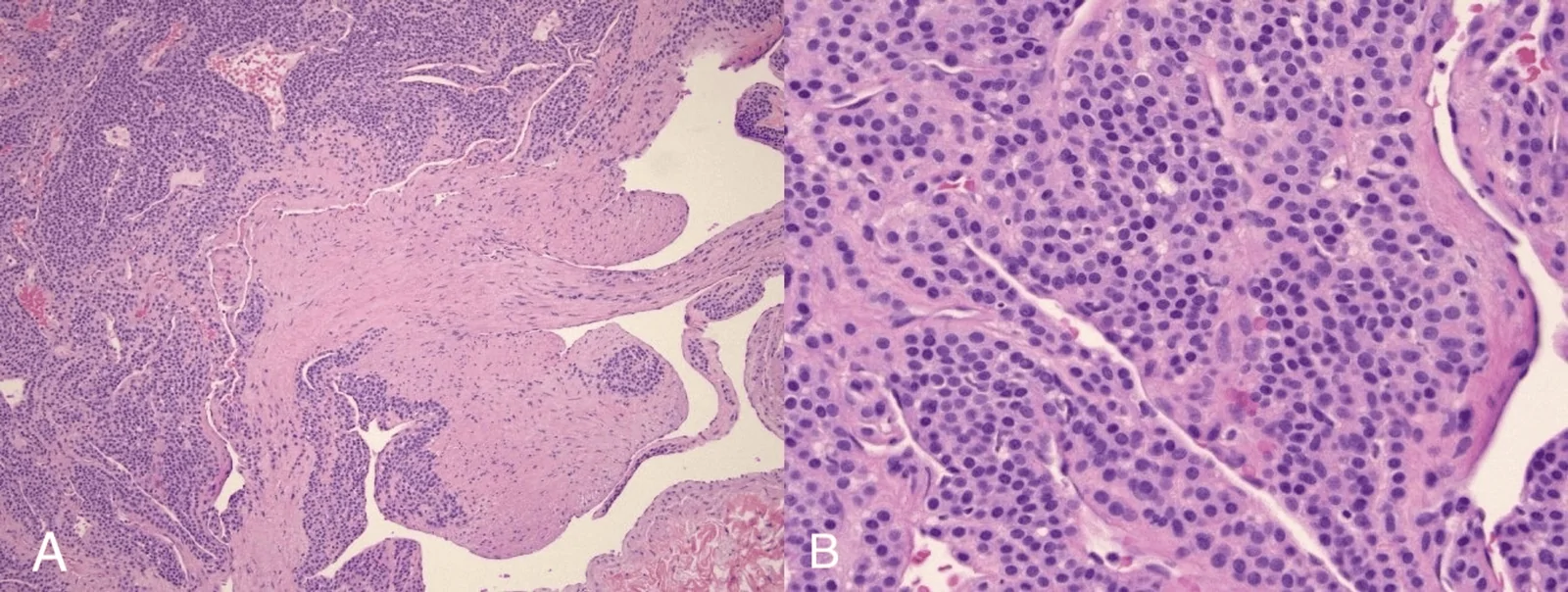
(A) Low-power and (B) high-power photomicrographs showing dilated vascular spaces surrounded by small, uniform polygonal glomus cells with eosinophilic cytoplasm and central nuclei.

The patient experienced immediate and complete resolution of his wrist pain following surgery. At one-year follow-up, he remained pain-free with no functional limitations and expressed high satisfaction with the outcome.

## Discussion

Glomus tumors are classified into three types based on their dominant histologic components, including solid (mainly glomus cells), glomangioma (mainly blood vessels), and glomangiomyoma (mainly smooth muscle cells). All are benign neoplasms arising from the glomus body, a thermoregulatory arteriovenous shunt most commonly found in the subungual region of the digits [2,3,5].

Although classically located beneath the fingernails, these tumors may rarely arise in extradigital locations such as the wrist, forearm, or trunk [5,6]. In atypical sites, diagnosis is often delayed due to their nonspecific presentation and the low index of suspicion among clinicians.

Patients typically present with localized pain and hypersensitivity. Unlike digital glomus tumors, which may present with the classic triad of pain, tenderness, and cold sensitivity, extradigital glomangiomas may lack distinctive features [5-7]. In this case, the patient’s longstanding wrist pain mimicked more common orthopedic conditions such as triangular fibrocartilage complex (TFCC) tears or extensor carpi ulnaris tendonitis [1]. The lesion's small size and deep subcutaneous location made palpation and diagnosis more challenging; however, the focal sensitivity and exquisite tenderness were important clues to its true identity. Most other soft tissue tumors, such as tenosynovial giant cell tumors and sarcomas, are not especially painful or tender when small [8].

Radiologic findings may aid in identification, with glomangiomas typically appearing as small, well-circumscribed T2 hyperintense lesions that enhance with contrast [9,10]. However, these findings can overlap with other benign and malignant tumors, requiring histologic sampling for definitive confirmation [5]. Superficial soft tissue masses below 3 cm in diameter are reasonable candidates for excisional biopsy, while larger masses are best approached in a staged fashion, typically with a needle biopsy prior to final excision [11]. Complete surgical excision remains the treatment of choice for glomus tumors and is usually curative [12]. In this patient, the symptoms were promptly relieved upon removal of the tumor.

This case highlights the need to consider uncommon soft tissue tumors in patients with persistent pain despite appropriate conservative management. When physical examination demonstrates an exquisitely painful lesion and MRI reveals a contrast-enhancing nodule in an atypical, nondigital location, the possibility of a glomus tumor, or one of its variants such as glomangioma, should be included in the differential diagnosis.

## Conclusions

In patients with chronic wrist pain unresponsive to standard treatments, uncommon etiologies such as tumors should be considered. Glomangioma is a rare tumor but might be suspected when there is exquisite focal tenderness. This case underscores the importance of advanced imaging, clinical vigilance, and surgical excision when unexpected soft tissue masses are identified as a possible source of pain. Careful surgical planning is vitally important since the differential diagnosis of even small masses may include sarcomas and other malignant tumors. Excisional biopsy is a reasonable consideration when the lesion is superficial and less than 3 cm in size. In this patient, surgical excision led to complete resolution of symptoms and excellent relief of pain, reaffirming the effectiveness of definitive surgical management for glomangiomas.
